# Tirzepatide compared with semaglutide and 10-year cardiovascular disease risk reduction in obesity: *post-hoc* analysis of the SURMOUNT-5 trial

**DOI:** 10.1093/ehjopen/oeaf117

**Published:** 2025-09-02

**Authors:** Mamas A Mamas, Harold Bays, Runjia Li, Navneet Upadhyay, Tanya Irani, Cagri Senyucel, Julia P Dunn, Hong Liu-Seifert

**Affiliations:** Keele Cardiovascular Research Group, School of Medicine, Keele University, Staffordshire ST5 5BG, UK; Louisville Metabolic and Atherosclerosis Research Center, 3288 Illinois Avenue, Louisville, KY 40213, USA; Eli Lilly and Company, 307 E Merrill, Indianapolis, IN 46285, USA; Eli Lilly and Company, 307 E Merrill, Indianapolis, IN 46285, USA; Eli Lilly and Company, 307 E Merrill, Indianapolis, IN 46285, USA; Eli Lilly and Company, 307 E Merrill, Indianapolis, IN 46285, USA; Eli Lilly and Company, 307 E Merrill, Indianapolis, IN 46285, USA; Eli Lilly and Company, 307 E Merrill, Indianapolis, IN 46285, USA

**Keywords:** Cardiovascular disease, Tirzepatide, Semaglutide

## Abstract

**Aims:**

Approximately two-thirds of obesity-related mortality is attributable to cardiovascular disease (CVD). The aim of this analysis is to examine predicted CVD risk reduction following weight loss in persons with obesity for primary prevention between tirzepatide and semaglutide, and projected CVD events that could be potentially prevented in the USA.

**Methods and results:**

SURMOUNT-5 was a Phase 3b, open-label, randomized trial conducted in participants with obesity and without Type-2 diabetes, comparing tirzepatide (10 or 15 mg) with semaglutide (1.7 or 2.4 mg) and administered via weekly subcutaneous injection. Predicted 10-year CVD risks were compared between treatments at baseline and up to 72 weeks post-treatment among participants without prior CVD. The impact of cardiovascular risk reduction was estimated as the projected preventable CVD events over 10 years for tirzepatide and semaglutide in the USA. The average predicted 10-year CVD risk score before treatment was 9.3%. Treatment with tirzepatide was associated with significantly greater reduction in predicted 10-year CVD risk compared with semaglutide (absolute reduction from baseline of 2.4% and 1.4%, respectively, *P* < 0.001). Translating risk reduction to the US population who met treatment eligibility criteria and without prior CVD (∼85 million), an estimated 2 million CVD events could be potentially prevented over 10 years after 72 weeks of tirzepatide treatment, vs. 1.15 million with semaglutide.

**Conclusion:**

In SURMOUNT-5, treatment with tirzepatide was associated with greater predicted 10-year CVD risk reduction compared with semaglutide. This *post hoc* analysis suggests tirzepatide treatment may provide greater benefit in primary prevention of CVD than semaglutide in people with obesity and overweight.

**Registration:**

ClinicalTrials.gov: NCT05822830

## Introduction

People with obesity or overweight have an increased risk of developing cardiovascular disease (CVD),^1,2^ with excess adiposity increasing the risk of CVD directly through negative effects on the endocrine and immune systems,^3,4^ and indirectly through the development of obesity-related complications, including type 2 diabetes (T2D), hypertension, dyslipidaemia, thrombosis or sleep disorders, which are CVD risk factors.^3^ Cardiovascular disease accounts for more than two-thirds of deaths in people with elevated body mass index (BMI).^5^

Historically, obesity management medications (OMMs) had a limited role in CVD due to modest weight reduction, lacking cardiovascular (CV) benefit, and even harm from older OMMs.^6^ New and emerging highly effective OMMs, such as tirzepatide and semaglutide, have contributed to major shifts in the practice of obesity care,^7^ with meaningful reductions in cardiovascular-related endpoints.^8–11^

In addition to BMI, waist circumference, used as a measure of abdominal obesity, has been highlighted as an independent CVD risk marker.^12^ The SURMOUNT-5 trial showed that treatment with tirzepatide (10 or 15 mg) was superior to semaglutide (1.7 or 2.4 mg) for reduction in weight and waist circumference in participants with obesity and without diabetes.^11^ After 72 weeks of treatment, participants in the tirzepatide-treated arm had a least-squares mean (LS mean) weight reduction of 20.2% vs. 13.7% in the semaglutide-treated arm (*P* < 0.001),^11^ adjusted for baseline weight, prediabetes status, BMI group (<35, ≥35 kg/m^2^), and sex.^11^ Regarding waist circumference, participants in the tirzepatide-treated arm had a change of −18.4 cm vs. −13.0 cm in the semaglutide-treated arm, from baseline to Week 72 (*P* < 0.001).

Research comparing the long-term CVD impact of OMMs such as tirzepatide and semaglutide is limited. Both semaglutide and tirzepatide are known to be beneficial in improving CVD-associated risk factors. Nevertheless, there has not been a head-to-head comparison for meaningful CV event rates between the two agents in a single trial in high-risk populations.^13,14^ In this analysis, we used patient-level longitudinal data from SURMOUNT-5 to determine the projected 10-year CVD risk between tirzepatide and semaglutide directly for the first time and estimated the number of potential CVD events that could be potentially prevented over a 10-year time frame in the USA. The aim of the study was to provide a direct comparison of the two treatments in predicted long-term cardiovascular benefit and its potential impact at the US population level based on National Health and Nutrition Examination Survey (NHANES) data.

## Methods

### Study design and population

SURMOUNT-5 is a Phase 3b, multicentre, open-label, randomized, active-controlled trial in adult participants with obesity and overweight with at least one obesity-related complication (ORC) [such as hypertension, dyslipidaemia, obstructive sleep apnoea (OSA), or CVD] and without T2D. Details of the SURMOUNT-5 study design have been published previously.^11^ Participants received treatment with either tirzepatide maximum tolerated dose (10 or 15 mg) or semaglutide maximum tolerated dose (1.7 or 2.4 mg) once weekly via subcutaneous injection for 72 weeks. The current analysis was a *post hoc* analysis of SURMOUNT-5 using patient-level data among those who did not have pre-existing CVD events at baseline to compare the change in predicted 10-year CVD risk from baseline up to 72 weeks between tirzepatide and semaglutide-treated groups.

Pre-existing CVD events at baseline were defined to include coronary artery disease, peripheral vascular disease, heart failure, and cerebrovascular disease. The demographic and baseline clinical characteristics were summarized by treatment group in participants without CVD events at baseline.

### Cardiovascular disease risk score calculation

The predicted 10-year CVD risk score was calculated using the BMI-based sex-specific risk equations (supplementary material online, *Methods*), which were developed from the Framingham Heart Study by D’Agostino *et al.*^15^ The general CVD events predicted in the model included coronary heart disease, stroke, peripheral artery disease, or heart failure.^15^ The risk model utilized factors of sex, age, BMI, systolic blood pressure (SBP), treatment for hypertension, smoking, and diabetes status. In this analysis, the diabetes status of participants after baseline was determined using glycated haemoglobin (HbA1c) and fasting serum glucose, following the same rule as the exclusion criteria of the trial, which were diagnoses of diabetes if HbA1c is ≥6.5%, or fasting glucose ≥126 mg/dL (≥7.0 mmol/L). The smoking status of participants was collected at baseline and assumed to remain constant throughout the study period.

### Statistical analyses

The estimated 10-year cardiovascular risk was compared between tirzepatide and semaglutide from treatment initiation to week 72 among participants without baseline CVD who completed study treatment (per-protocol, complete-case analysis; no imputation). The treatment differences in risk reduction were analysed using analysis of covariance (ANCOVA) models including treatment, baseline 10-year CVD risk score, prediabetes status, BMI group (<35, ≥35 kg/m^2^), sex, and age. To approximate the marginal hazard ratio (HR), the ratio of the negative logarithms of the group-level predicted 10-year survival probabilities was used, under the proportional hazard assumption.^16^ This approach utilized the relationship between survival probability and cumulative hazards, and targeted a marginal (population-averaged) contrast.^16,17^ Additional subgroup analyses were conducted to assess the treatment differences stratified by sex, race, and baseline BMI group. To translate the resulting risk reduction effect to population-level impact, this study estimated the OMM treatment-eligible population of the USA. Individuals were eligible if they had a BMI ≥30 kg/m^2^ or BMI ≥27 kg/m^2^ with at least one obesity-related complication (i.e. dyslipidaemia, OSA, or hypertension), and if they did not have T2D or pre-existing CVD events (see supplementary material online, *Table S1*).

### US population projection using National Health and Nutrition Examination Survey data

The NHANES^18^ was used to derive the US population size that was eligible for tirzepatide and semaglutide, as stated above. NHANES is a US National Survey that is administered by the US National Center of Health Statistics periodically to measure the health and nutrition of the US population and is representative of the US population. This study used the latest NHANES survey of 2021–23 to identify participants that met the study criteria (see Supplementary material online, *Table S1*) and used sample weighting to generalize to the US population in millions. To identify the population without diabetes, those with evidence of diabetes identified through self-reported response to the following questions were excluded (i) HbA1c of 6.5% or greater (ii) fasting glucose ≥126 mg/dL, (iii) Health Care Provider confirmed diabetes mellitus (DM), or (iv) taking DM medication or insulin. To identify survey respondents without prior CVD, individuals with self-reported history of congestive heart failure, coronary heart disease, angina/angina pectoris, heart attack, and stroke were excluded. NHANES 2021–23 does not include the variables to identify OSA; therefore, estimates for the population with overweight and with one ORC do not represent those with OSA.

The impact of tirzepatide and semaglutide on the cardiovascular risk reduction on the population level in the USA is projected as the preventable CVD events over 10 years if the eligible population without CVD history is treated with tirzepatide or semaglutide, respectively, calculated for each treatment by multiplying 10-year CV risk reduction derived for tirzepatide and semaglutide respectively with the treatment-eligible populations without prior CVD events.

## Results

Overall, 751 participants were randomized, and 80.2% completed the 72-week treatment.^11^ In the 576 participants who did not have pre-existing CVD and completed 72-week treatment (see Supplementary material online, *Figure S1*), the average predicted 10-year CVD risk score before treatment was 9.3%. Participants were mostly female (65.8%), White (75.3%), and with a mean age of 44.8 ± 11.9 years and BMI (kg/m^2^) of 39.5 ± 7.55. Of these participants, 6.9% were smokers, 37.2% were on treatment for hypertension, and 57.5% had prediabetes (*Table 1*). The baseline characteristics are comparable between treatment groups.

**Table 1:** SURMOUNT-5 Baseline characteristics (population without cardiovascular disease)

	Overall	Semaglutide 2.4 mg or MTD	Tirzepatide 15 mg or MTD
	(*N* = 576)	(*N* = 284)	(*N* = 292)
Sex			
Female	379 (65.8%)	188 (66.2%)	191 (65.4%)
Male	197 (34.2%)	96 (33.8%)	101 (34.6%)
Age, years	44.8 (11.9)	44.8 (11.8)	44.7 (12.0)
Race			
Asian	14 (2.4%)	6 (2.1%)	8 (2.7%)
Black or African American	114 (19.8%)	57 (20.1%)	57 (19.5%)
Multiple	8 (1.4%)	5 (1.8%)	3 (1.0%)
White	434 (75.3%)	216 (76.1%)	218 (74.7%)
American Indian or Alaska Native	6 (1.0%)	0 (0%)	6 (2.1%)
Ethnicity			
Hispanic or Latino	146 (25.3%)	75 (26.4%)	71 (24.3%)
Not Hispanic or Latino	430 (74.7%)	209 (73.6%)	221 (75.7%)
Current smoker	40 (6.9%)	21 (7.4%)	19 (6.5%)
Treatment for hypertension	214 (37.2%)	107 (37.7%)	107 (36.6%)
Lipid-lowering medications	119 (20.7%)	60 (21.1%)	59 (20.2%)
Prediabetes	331 (57.5%)	162 (57.0%)	169 (57.9%)
Weight (kg)	113 (25.8)	113 (26.4)	114 (25.2)
BMI (kg/m^2^)	39.5 (7.6)	39.4 (7.7)	39.6 (7.4)
Waist circumference (cm)	118 (16.6)	118 (17.3)	118 (16.0)
Total cholesterol (mg/dL)	190 (35.9)	191 (33.8)	188 (37.9)
HDL (mg/dL)	49.8 (13.0)	50.4 (13.2)	49.1 (12.8)
LDL (mg/dL)	114 (31.4)	115 (30.7)	114 (32.2)
Triglycerides (mg/dL)	130 (66.5)	129 (63.6)	130 (69.3)
HbA1c, %	5.63 (0.36)	5.64 (0.38)	5.61 (0.33)
Fasting serum glucose, mmol/L	5.26 (0.56)	5.29 (0.53)	5.24 (0.59)
Systolic blood pressure, mmHg	125.8 (13.1)	125.9 (12.6)	125.6 (13.7)
Diastolic blood pressure, mmHg	81.3 (8.3)	81.6 (8.2)	81.0 (8.4)
eGFR (mL per min 1.73 m^2^)	99.7 (17.1)	99.5 (16.9)	99.8 (17.3)

BMI, body mass index; eGFR, estimated glomerular filtration rate.

**Figure 1:**
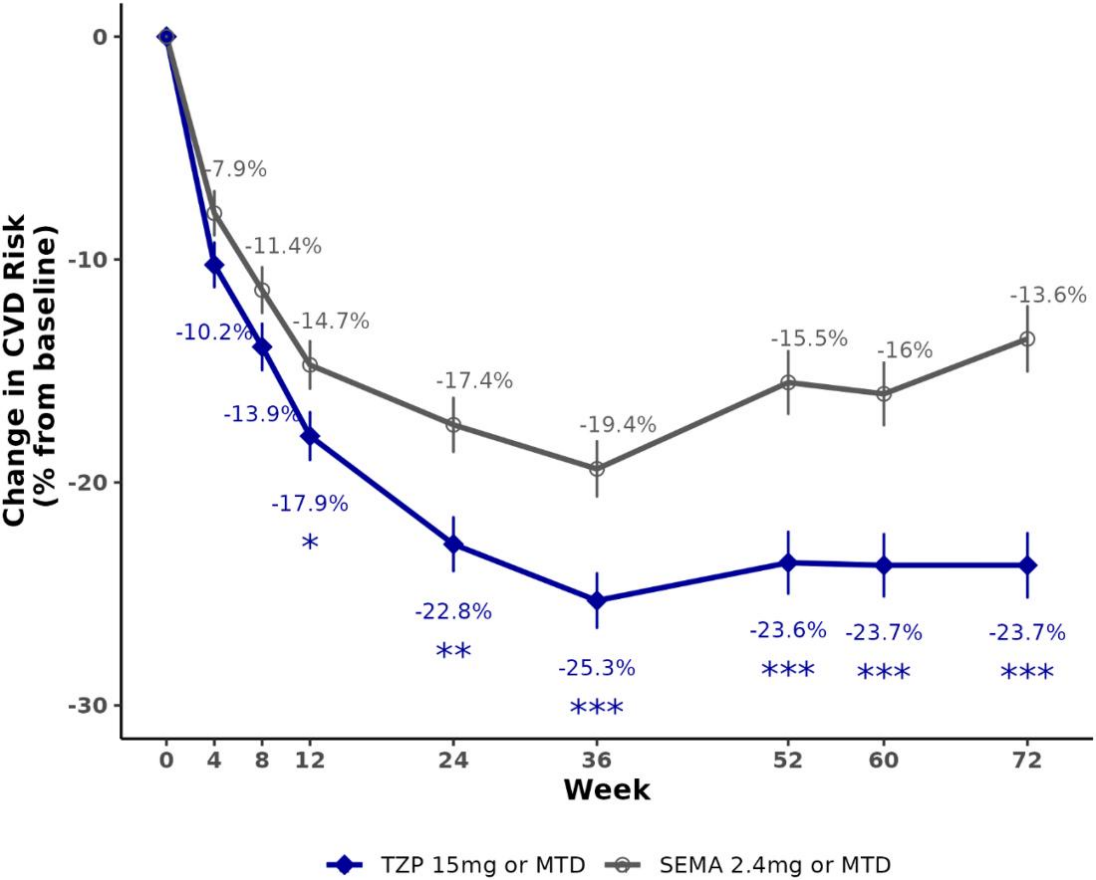
Percent change from baseline in cardiovascular risk (%), in tirzepatide vs. semaglutide. Data are least-square mean for per cent (%) change from baseline. Error bars indicate standard error. MTD, maximum tolerated dose; SEMA, semaglutide; TZP, tirzepatide.

Treatment with tirzepatide was associated with a significantly greater predicted 10-year CVD risk reduction compared with semaglutide at 72 weeks. *Table 2* shows the absolute reduction from baseline to 72 weeks of 2.36% in the tirzepatide-treated group, and 1.35% in the semaglutide-treated group (*P* < 0.001). Further, tirzepatide was associated with a 23.72% relative reduction from baseline in 10-year CVD risk compared with a 13.56% relative reduction from baseline in semaglutide (*P* < 0.001). The marginal HR was estimated as 0.87, reflecting an ∼13% reduction in hazard comparing tirzepatide to semaglutide after 72 weeks of treatment. When evaluating over the course of the 72 weeks, the greater treatment effect of tirzepatide compared with semaglutide in CVD risk reduction was observed as early as 12 weeks of treatment (*Figure 1*).

**Table 2:** Estimated 10-year cardiovascular disease risk

		Tirzepatide (15 mg or MTD) (*N* = 292)			Semaglutide (2.4 mg or MTD) (*N* = 284)		
		CVD risk (%)		CFB in CVD risk	CVD risk (%)		CFB in CVD risk
	*N*	Baseline	72 W^a^	(LSM; %)^a^	Baseline	72 W^a^	(LSM; %)^a^
Overall	576	9.01	6.89	−2.36	9.50	7.90	−1.35
Female	379	5.81	4.24	−1.68	6.04	4.80	−1.12
Male	197	15.08	11.93	−3.74	16.28	13.96	−1.71
Non-White	142	9.21	5.94	−1.59	5.71	6.21	−1.32
White	434	8.95	7.25	−2.56	10.69	8.40	−1.42
BMI <35 kg/m^2^	175	8.81	6.84	−2.10	9.05	7.35	−1.58
BMI ≥35 kg/m^2^	401	9.10	6.93	−2.46	9.72	8.14	−1.25

^a^Only subjects with non-missing baseline value and 72-week value of the response variable were included in the analysis. ANCOVA model for baseline measures: Variable = Treatment + Baseline 10-year CVD Risk + Sex + Baseline BMI Group + Prediabetes + Age.

BMI, body mass index; CFB, change from baseline; CVD, cardiovascular disease; LSM, least-squares mean; MTD, maximum tolerated dose; W, week.

**Figure 2:**
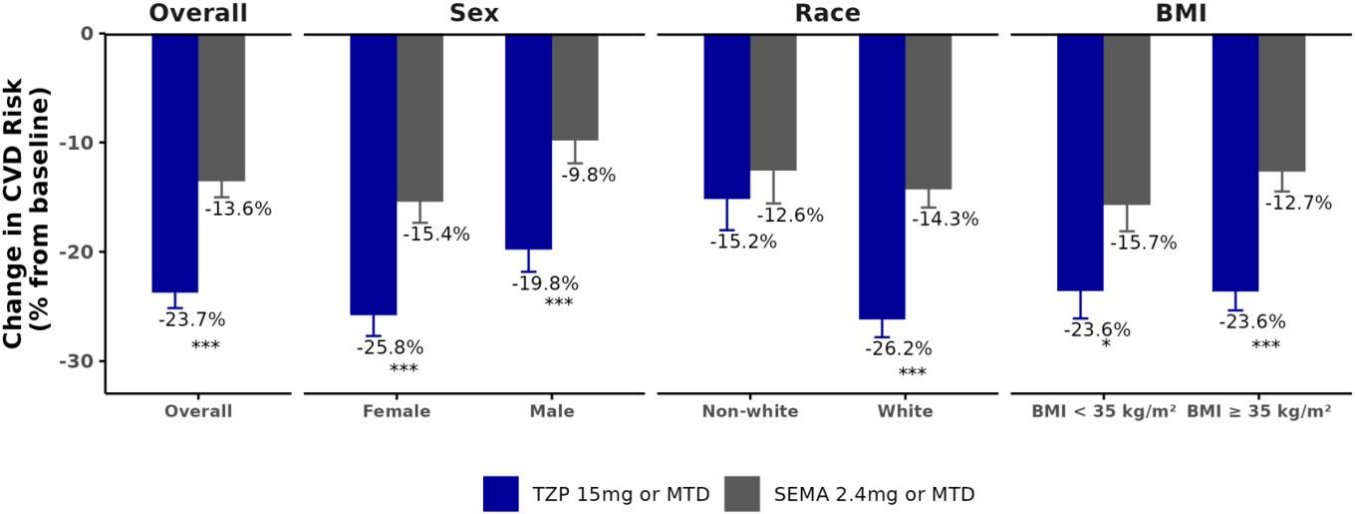
Percent change from baseline (PCFB) in cardiovascular risk, % in tirzepatide vs. semaglutide, by baseline subgroup. BMI, body mass index; MTD, maximum tolerated dose; SEMA, semaglutide; TZP, tirzepatide.

Across treatment groups, females had lower CVD risks than males at baseline. At Week 72, the predicted 10-year CVD risk was reduced to 4.24% in females (1.68% absolute reduction) vs. 11.93% in males (3.74% absolute reduction) in the tirzepatide-treated participants, and to 4.80% in females (1.12% absolute reduction) vs. 13.96% in males (1.71% absolute reduction) in semaglutide-treated participants (*Table 2*). Additional subgroup analyses by race (White vs. non-White) and baseline BMI category showed consistent treatment effect of tirzepatide over semaglutide in reducing the 10-year CVD risk (*Table 2* and *Figure 2*).

**Figure 3:**
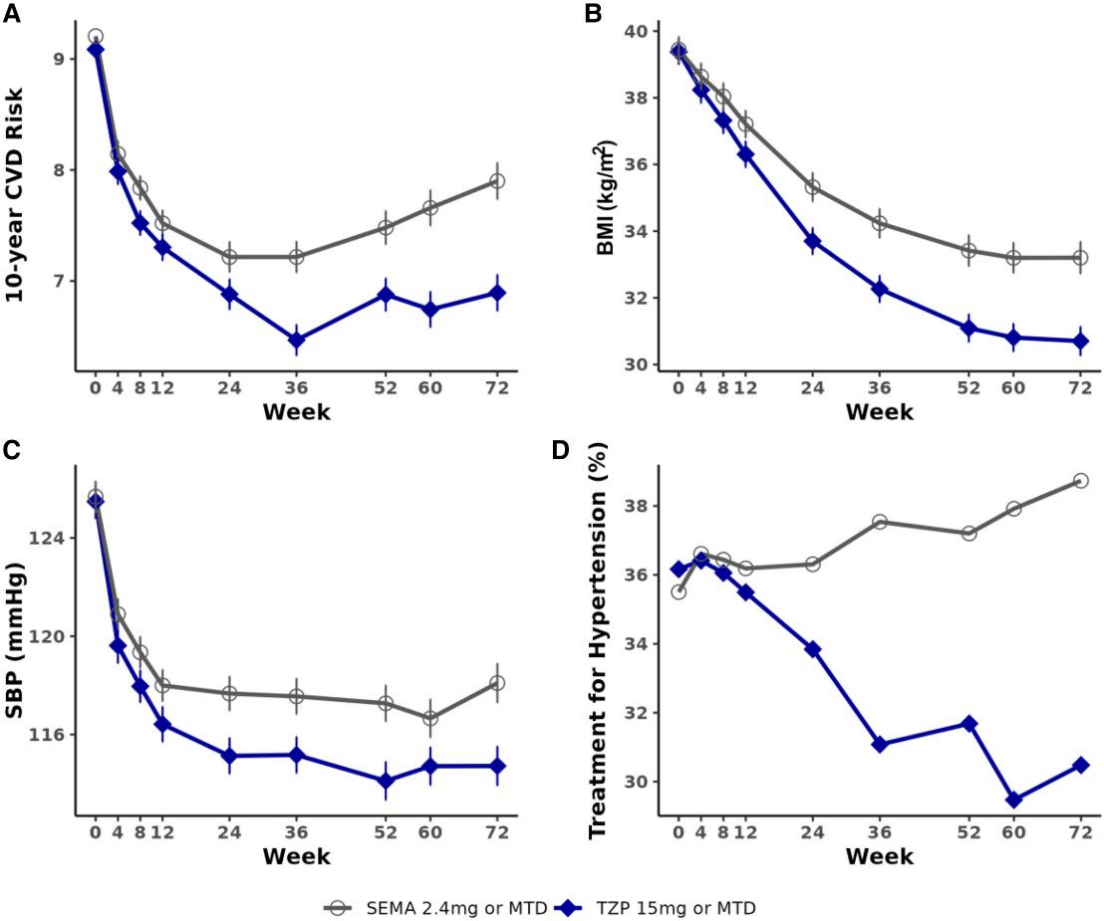
Predicted 10-year cardiovascular risk and risk factors in tirzepatide vs. semaglutide from baseline to Week 72. Data are least-square mean for post-baseline 10-year cardiovascular disease risk, and observed mean for baseline 10-year cardiovascular disease risk, body mass index, systolic blood pressure, and the proportion of under treatment for hypertension (%). Error bars indicate standard error. MTD, maximum tolerated dose; SEMA, semaglutide; TZP, tirzepatide.

Longitudinal trajectories of the predicted 10-year cardiovascular risk and its contributing risk factors—BMI, SBP, and use of antihypertensive medication—are shown by treatment group (*Figure 3*). Participants receiving tirzepatide exhibited greater reductions in BMI and SBP over time than semaglutide. A decrease in the proportion of participants on antihypertensive treatment was also observed in the tirzepatide group. At week 72, one tirzepatide participant met the criteria for diabetes [HbA1c ≥6.5% or fasting plasma glucose ≥126 mg/dL (≥7.0 mmol/L)]. Further, data on lipid parameters over time showed consistent treatment trajectories in addition to the risk factors included in the risk equations (see Supplementary material online, *Figure S2*).

The results on the predicted 10-year CVD risk reduction were translated into the estimated US population who met treatment eligibility criteria (and without prior CVD), contributing to a population of ∼85 million potential patients. Based on risk reduction results, if all the eligible population received treatment with these OMMs and the 72-week effect persisted on treatment, an estimated 2.0 million CVD events could be potentially prevented with tirzepatide over 10 years, compared with 1.15 million with semaglutide.

## Discussion

Tirzepatide was associated with a greater reduction in predicted 10-year CVD risk compared with semaglutide in participants with obesity and without diabetes or pre-existing CVD. Significant treatment effects were seen as early as 12 weeks after randomization and maintained to the end of the study. This greater risk reduction translated to an estimated 0.86 million additional primary CVD events potentially prevented over 10 years with tirzepatide compared with semaglutide in the eligible US population.

New generation incretin-based medications such as tirzepatide and semaglutide have been shown to be highly effective in weight reduction in people with obesity or overweight.^7^ While studies have reported beneficial effects in cardiovascular outcomes in populations with T2D,^19–23^ data to understand the long-term health benefits, such as CVD risk reduction, in patients with obesity are limited.^24,25^ It has been shown that more substantial weight reduction in individuals with overweight or obesity is associated with a decreased CVD risk.^26^ It is of interest to understand the treatment effect on the primary prevention of CVD in patients with obesity and without pre-existing CVD.

The SELECT trial, which included 17 604 participants over 45 years old with established CVD, a BMI of 27 kg/m^2^ or higher and without T2D, demonstrated that semaglutide reduced three-point major adverse cardiovascular events by 20% compared with placebo over a mean follow-up duration of 39.8 months.^27^ However, it was a secondary prevention study, and there are no data available to evaluate the primary prevention of CVD events in the relevant population.

The ongoing SURMOUNT-MMO trial (an outcome study of tirzepatide on reduction of morbidity and mortality in adults with obesity; ClinicalTrials.gov number, NCT05556512) includes both primary and secondary prevention cohorts, providing data to the long-term health benefits related to tirzepatide treatment, including the impact on CVD events. While the results are pending, consistent data show improvement in several cardiometabolic risk factors with tirzepatide.^9,11^

The results are consistent between these current analyses of SURMOUNT-5 data and those by Wong *et al.*^13,14^ particularly in the treatment effect of tirzepatide vs. semaglutide on CVD risk reduction. The estimated baseline 10-year CVD risk in the SURMOUNT-5 data is slightly lower than in the work by Wong *et al.*, due to the lower proportion of males (34% and 50%, respectively) and a younger population (mean age 44.8 and 47.3, respectively). While the absolute 10-year CVD risk reduction is similar between this work and Wong *et al.* for both tirzepatide and semaglutide, this work allowed the direct comparison of the two treatment groups in one single randomized and controlled study.

There are a number of strengths in the current analysis. The most relevant is the direct comparison of tirzepatide and semaglutide in the predicted 10-year CVD risk reduction facilitated by the only head-to-head study of the two leading OMMs. Secondly, the 10-year CVD risk scores are calculated using the patient-level data at all time points in the study. Furthermore, the Framingham predicted 10-year CVD risk equations are well accepted and widely used in clinical practice.^28^ These risk equations are also applied in cost effectiveness modelling for healthcare coverage evaluations, such as by the Institute for Clinical and Economic Review (ICER).^29^

A difference in predicted cardiovascular risk reduction between treatment groups emerged as early as Week 12, preceding the full extent of clinically relevant weight loss. This temporal pattern raises the possibility of an independent treatment effect on cardiovascular risk beyond weight reduction. Tirzepatide is a dual agonist of glucose-dependent insulinotropic polypeptide (GIP) and glucagon-like peptide-1 (GLP-1) receptors, whereas semaglutide is a GLP-1 receptor agonist. Studies have shown that GLP-1 RA improve cardiovascular outcomes, and preclinical and translational studies have demonstrated mechanisms that include direct effects on the heart and vasculature and indirect effects via weight loss, glycaemic control, and anti-inflammatory actions.^30^ While the role of GIP on the cardiovascular system is less well-defined, preclinical studies showed that both GIP and GLP-1 RA reduce atherosclerotic burden and other pathologic features of CVD, including anti-inflammatory actions.^30^ Further exploration is warranted to disentangle potential direct cardiometabolic effects of tirzepatide from those mediated through weight reduction.

The lower predicted cardiovascular risk observed among female participants than males is consistent with existing literature^31,32^ and reflects the sex-specific structure of the Framingham risk equation and distribution of CV risk factors. To our knowledge, limited data are available from prior studies directly comparing sex-specific effects of tirzepatide or semaglutide on cardiovascular risk markers in primary prevention populations. Further investigation in larger, adequately powered cohorts is warranted to determine whether the treatment effect and mechanism differ meaningfully by sex.

One of the limitations of this work is that the Framingham 10-year CVD risk equations were developed based on a predominantly White population. We have conducted sensitivity analyses by applying the American Heart Association (AHA) Predicting Risk of CVD Events (PREVENT) risk formula based on diverse population, which produced directionally consistent treatment effect.^33^ Further, while the subgroup analyses based on race showed consistent treatment effect in White vs. non-White, further exploration with a larger sample of participants with racial diversity is warranted. The potential ‘prevented events’ was a scenario-based projection rather than an observed outcome. In SURMOUNT-5, while the vast majority of semaglutide participants achieved 2.4 mg, both 1.7 and 2.4 mg doses were allowed. As only 2.4 mg is an approved maintenance dose in certain regions, the findings may not be generalizable in routine clinical practice in all regions globally.^34^ Lastly, as smoking status was only collected at baseline and smokers may give up smoking after treatment, this study may underestimate the overall CVD risk reduction.

The prevalence of obesity is increasing globally, affecting 650 million adults.^35^ In particular, 40.3% of adults live with obesity in the USA.^36^ It is well known that obesity and obesity-related CVD are associated with significant costs to societies.^37^ Furthermore, the Presidential Advisory from the AHA has positioned obesity as a central and unifying driver of the cardiovascular-kidney-metabolic (CKM) syndrome, which reflects the interconnected pathophysiology of CVD, chronic kidney disease (CKD), and metabolic disorders like T2D.^38^ Excess adiposity/obesity acts as both an initiating factor and an amplifier of metabolic dysfunction, promoting insulin resistance, systemic inflammation, and dyslipidaemia—all of which contribute to CVD and CKD. Cardiovascular-kidney-metabolic syndrome carries serious clinical consequences, including premature death, multi-organ dysfunction, and substantial healthcare costs.^38,39^ According to the CKM Presidential Advisory, obesity and overweight contribute significantly to these burdens, with estimated annual direct costs of almost $500 billion and indirect productivity losses reaching $1.2 trillion in the USA.^38,39^ Addressing obesity through lifestyle interventions, pharmacotherapy, and in some cases bariatric surgery is emphasized as a cornerstone strategy for preventing and managing CKM syndrome.

Given the global obesity trend, and the availability of the new generation of OMMs, it is important to understand the impact of weight reduction on long-term health benefits at the population level. The association between tirzepatide vs. semaglutide and CVD risk reduction is consistent with previous real-world evidence studies.^13,20,21,14,40^ Based on this analysis, there is the potential that tirzepatide might have a benefit vs. semaglutide in the primary prevention of CVD events.

## Conclusions

The ultimate health benefit of weight reduction should be long-term health improvement. In this *post hoc* analysis of the SURMOUNT-5 study, treatment with tirzepatide was associated with a greater predicted 10-year CVD risk reduction compared with semaglutide. This suggests that tirzepatide treatment may play a greater role than semaglutide in the primary prevention of CVD in people with obesity.

## Supplementary Material

oeaf117_Supplementary_Data

## Data Availability

Eli Lilly and Company provides access to all individual participant data collected during the trial, after anonymization, with the exception of pharmacokinetic or genetic data. Data are available to request 6 months after the indication studied has been approved in the USA and EU and after primary publication acceptance, whichever is later. No expiration date for data requests is currently set once the data are made available. Access is provided after a proposal has been approved by an independent review committee identified for this purpose and after receipt of a signed data sharing agreement. Data and documents, including the study protocol, statistical analysis plan, blank or annotated case report forms, will be provided in a secure data sharing environment. For details on submitting a request, see the instructions provided at www.vivli.org.

## Lead author biography

Professor Mamas is trained in Medicine at Oxford University. He is a Professor of Cardiology (Keele University) and a practising interventional cardiologist. He is a member of AHA CLCD Cardiovascular Interventions Science Committee, board member of ESC Cardio-Oncology, and lead at British Cardiovascular Intervention Society National Registry. He serves as Associate Editor of *Circulation: Cardiovascular Interventions*. He is Clinical Director of the Centre for Prognosis Research, whose research focuses on electronic healthcare data to inform diagnosis, treatment, and clinical outcomes of real-world patients with cardiovascular disease. He has published over 1000 peer-reviewed manuscripts, cited over 58 000 times, with a *h*-index of 98.
