# Supplementary material for: Tirzepatide compared with semaglutide and 10-year cardiovascular disease risk reduction in obesity: post-hoc analysis of the SURMOUNT-5 trial
Source: Eur Heart J Open. 2025 Sep 2;5(5):oeaf117. doi: 10.1093/ehjopen/oeaf117 (PMC12448458; doi:10.1093/ehjopen/oeaf117)
Supplement: oeaf117_Supplementary_Data [file oeaf117_supplementary_data.docx]

**SUPPLEMENTAL**

Tirzepatide compared with semaglutide and 10-year cardiovascular disease risk reduction in obesity: *post hoc* analysis of the SURMOUNT-5 trial

**CONTENTS**

1. Supplemental Method
2. Figure S1
3. Table S1
4. Figure S2
5. **Supplemental Method**

The 10-year CVD risk (%) are predicted using BMI-based calculations by D'Agostino et al. 2008 [15]:
 Female:

10-year CVD Risk =1-0.94833 ^exp (Risk Part-26.0145)^,

where:

Risk Part = 2.72107*1og (Age) + 0.51125*log (BMI) + β_SBP_ *log (SBP) + 0.61868*Smoker +0.77763*Diabetes,

β_SBP_ for treated by anti-hypertensive medicine =2.88267; β_SBP_ for untreated = 2.81291.

Male:

10-year CVD Risk = 1-0.88431^exp (Risk Part-23.9388)^,

where:

Risk Part = 3.11296*log (Age) +0.79277*log (BMI) + β_SBP_*log (SBP) +0.70953*Smoker +0.53160*Diabetes,

β_SBP_ for treated by anti-hypertensive medicine = 1.92672; β_SBP_ for untreated = 1.85508

1. **Figure S1**


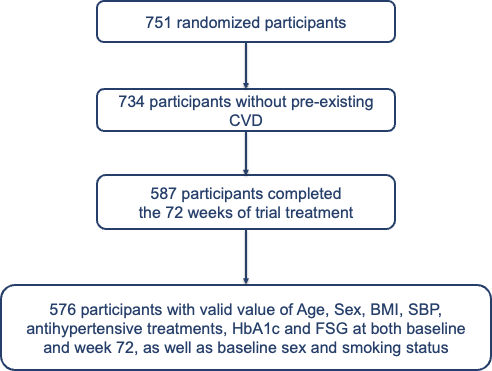


**FIGURE S1: Participants data for SURMOUNT-5 Analysis on CVD Risk.**

1. **TABLE S1: Population Attrition table for treatment eligible population and without prior CVD for US based on NHANES survey (2021-2023).**

|  | **Criteria** | **Population** |
| --- | --- | --- |
| A | US Adult Population | 253.8M |
| B | Population with 27kg/m^2^ ≤BMI<30kg/m^2^ | 45.6M |
| C | Proportion of those with 27 kg/m^2^ ≤ BMI<30kg/m^2^, at least one ORC | 52% |
| D | B X C | 23.8M |
| E | Population with Obesity | 99.6M |
| F | D+E | 123.4M |
| G | Among D, Proportion of those with no T2D & no prior CVD | 69% |
| H | Final eligible population for analysis | 85M |

Notes: ORC (obesity related complications): Hypertension, Dyslipidemia, T2DM, CVD at any time. Prior CVD, individuals with self-reported history of congestive heart failure, coronary heart disease, angina/angina pectoris, heart attack, and stroke.

1. **Figure S2**


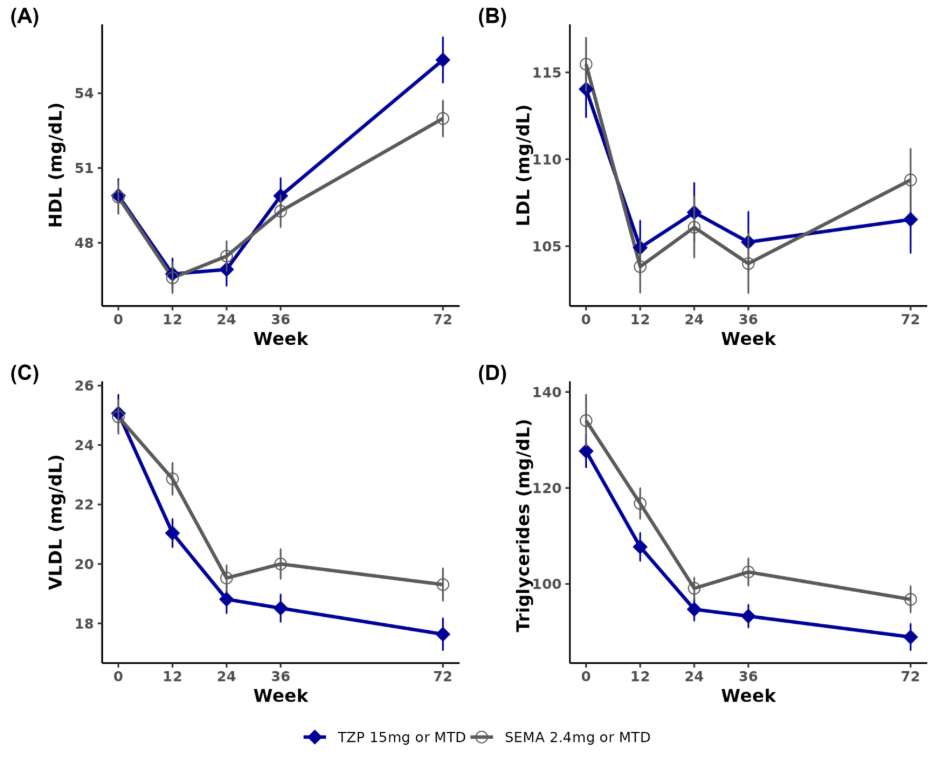


**Figure S2. Lipid parameters in tirzepatide vs semaglutide from baseline to 72 weeks.**

Note: Data are observed mean. Error bars indicate standard error. HDL = High-Density Lipoprotein; LDL = Low-Density Lipoprotein; MTD = Maximum tolerated dose; SEMA = Semaglutide; TZP = Tirzepatide; VLDL = Very-Low-Density Lipoprotein.
